# Supplementary material for: Characterization of Tumor-Infiltrating Lymphocyte-Derived Atypical TCRs Recognizing Breast Cancer in an MR1-Dependent Manner
Source: Cells. 2024 Oct 16;13(20):1711. doi: 10.3390/cells13201711 (PMC11506377; doi:10.3390/cells13201711)
Supplement: Supplementary file 1 [file cells-13-01711-s001.zip › cells-3145117-supplementary.pdf]

Supplementary information for

Characterization of Tumor-Infiltrating Lymphocyte-Derived Atypical TCRs Recognizing Breast Cancer in an MR1-Dependent Manner

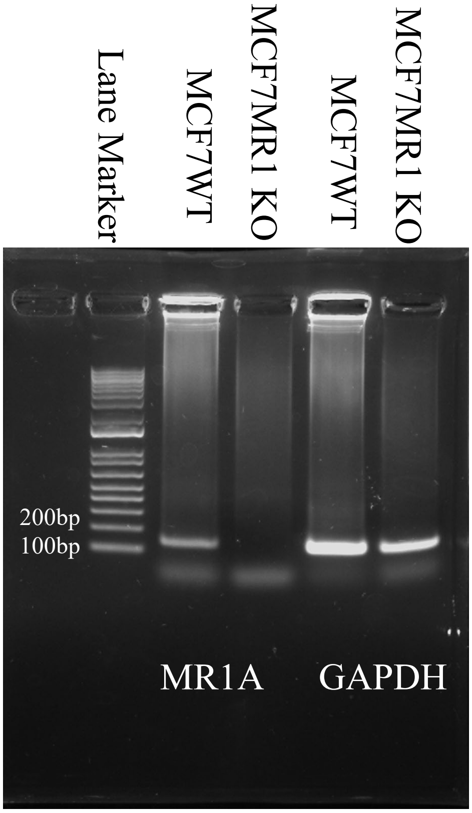

Figure S1. Expression of the MR1 gene in MCF7 cells. RT-PCR was performed with RNA extracted separately from MCF7WT and MCF7 ΔMR1 cells. GAPDH was used as control. Gel eletrophoresis of PCR products was performed. The sizes of the amplified bands are indicated.

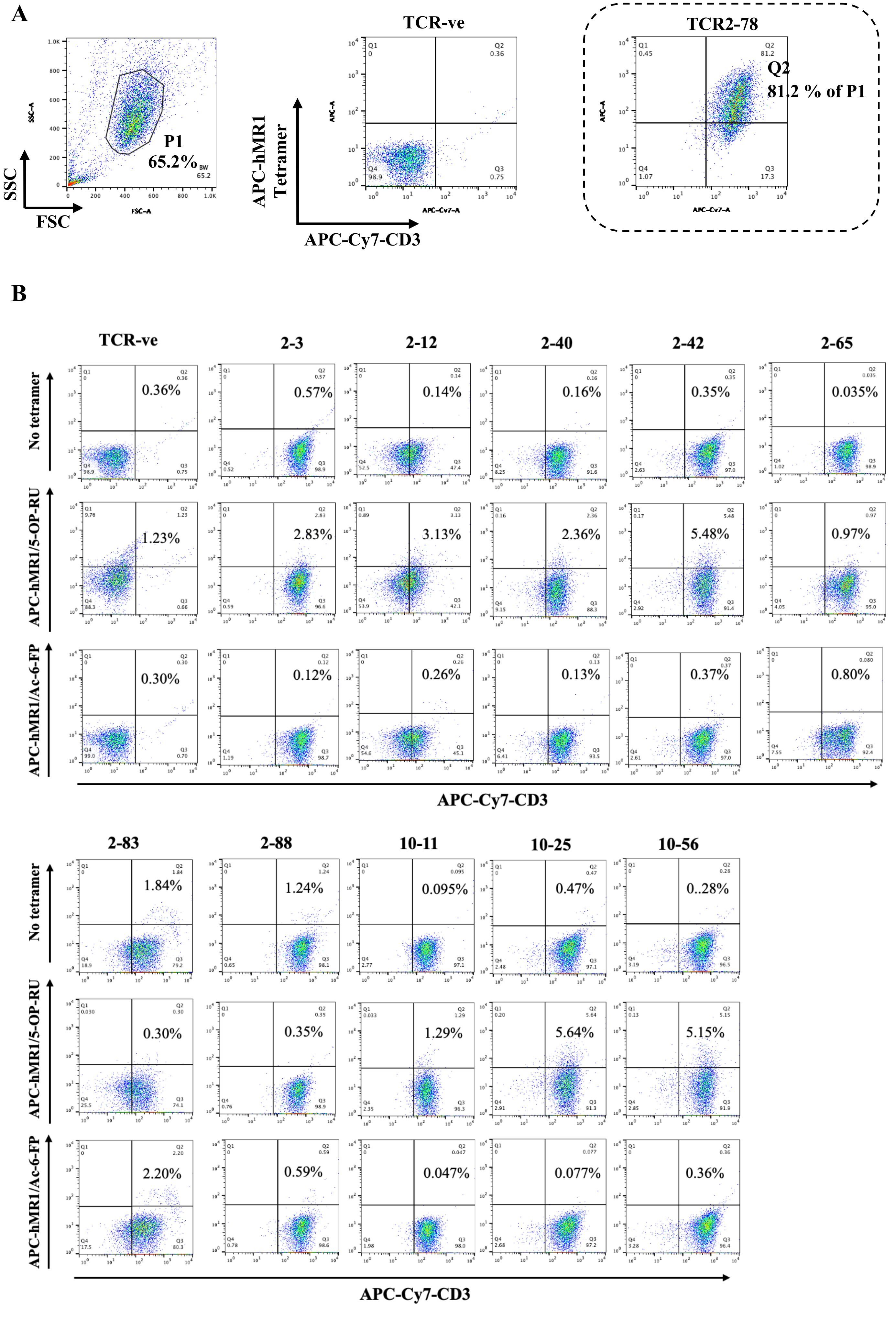

A
SSC
FSC
P1
65.2%
TCR-ve
APC-hMR1 Tetramer
APC-Cy7-CD3
TCR2-78
Q2
81.2 % of P1
B
TCR-ve
2-3
2-12
2-40
2-42
2-65
No tetramer
0.36%
0.57%
0.14%
0.16%
0.35%
0.035%
APC-hMR1/5-OP-RU
1.23%
2.83%
3.13%
2.36%
5.48%
0.97%
APC-hMR1/Ac-6-FP
0.30%
0.12%
0.26%
0.13%
0.37%
0.80%
APC-Cy7-CD3
2-83
2-88
10-11
10-25
10-56
No tetramer
1.84%
1.24%
0.095%
0.47%
0..28%
APC-hMR1/5-OP-RU
0.30%
0.35%
1.29%
5.64%
5.15%
APC-hMR1/Ac-6-FP
2.20%
0.59%
0.047%
0.077%
0.36%
APC-Cy7-CD3

Figure S2. MR-1 specificity of HLA-independent TCRs. (A) Gating strategy for analysis of CD3+tetramer+ cells. Cells were stained with APC-conjugated MR1 tetramers (hMR1/6-FP and hMR1/5-OP-RU) and APC-Cy7-conjugated mouse CD3 monoclonal antibody (mAb). The gate of live BW-hCD8αβ+ cells was set using side scatter and forward scatter (P1). The percentage of CD3+tetramer+ cells was indicated in right upper quadrant Q2. (B) BW-hCD8αβ+ (BW) cells transfected with MR1-restricted TCRs from two breast cancer patients and stained with APC-conjugated MR1 tetramers (hMR1/6-FP and hMR1/5-OP-RU) and APC-Cy7-conjugated mouse CD3 monoclonal antibody (mAb). Right upper quadrant of dot plots is showing CD3+tetramer+ cells. TCR negative (TCR-ve) BW cells were used as negative control. Plots are representative of two independent experiments. hMR1, Human MR1, 6-FP, 6-formylpterin and 5-OP-RU *,5-(2-oxopropylideneamino)-6-D-ribitylaminouracil*

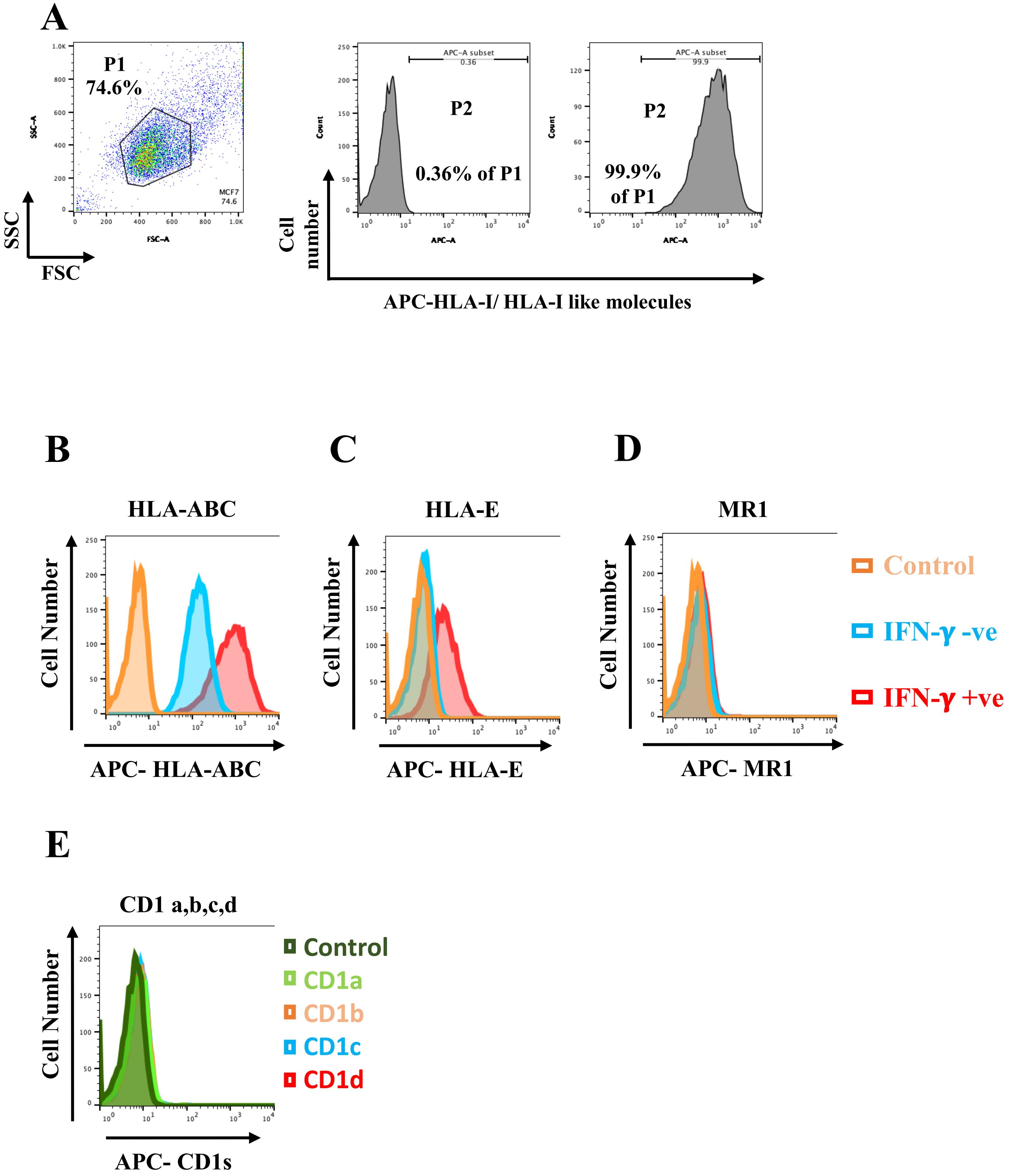

Figure S3. Characterization of unconventional HLA-class I like molecules on MCF7; breast cancer cell line. MCF7 cells were stained with APC- conjugated mAbs to analyze cell surface expression of HLA-I or unconventional HLA-I like molecules. (A) The gate of live MCF7 cells was set using side scatter and forward scatter (P1) and the percentages of APC+ cells were indicated in P2. Histograms showing the surface expression of HLA-A,B,C (*B*), HLA-E (*C*), MR1 expression (*D*) on MCF7 cells with/without stimulation with hIFN-γ for 48 hours. (*E*) Expression of CD1 molecules on MCF7 cells after stimulation with hIFN-γ for 48 hours. All experiments performed twice.

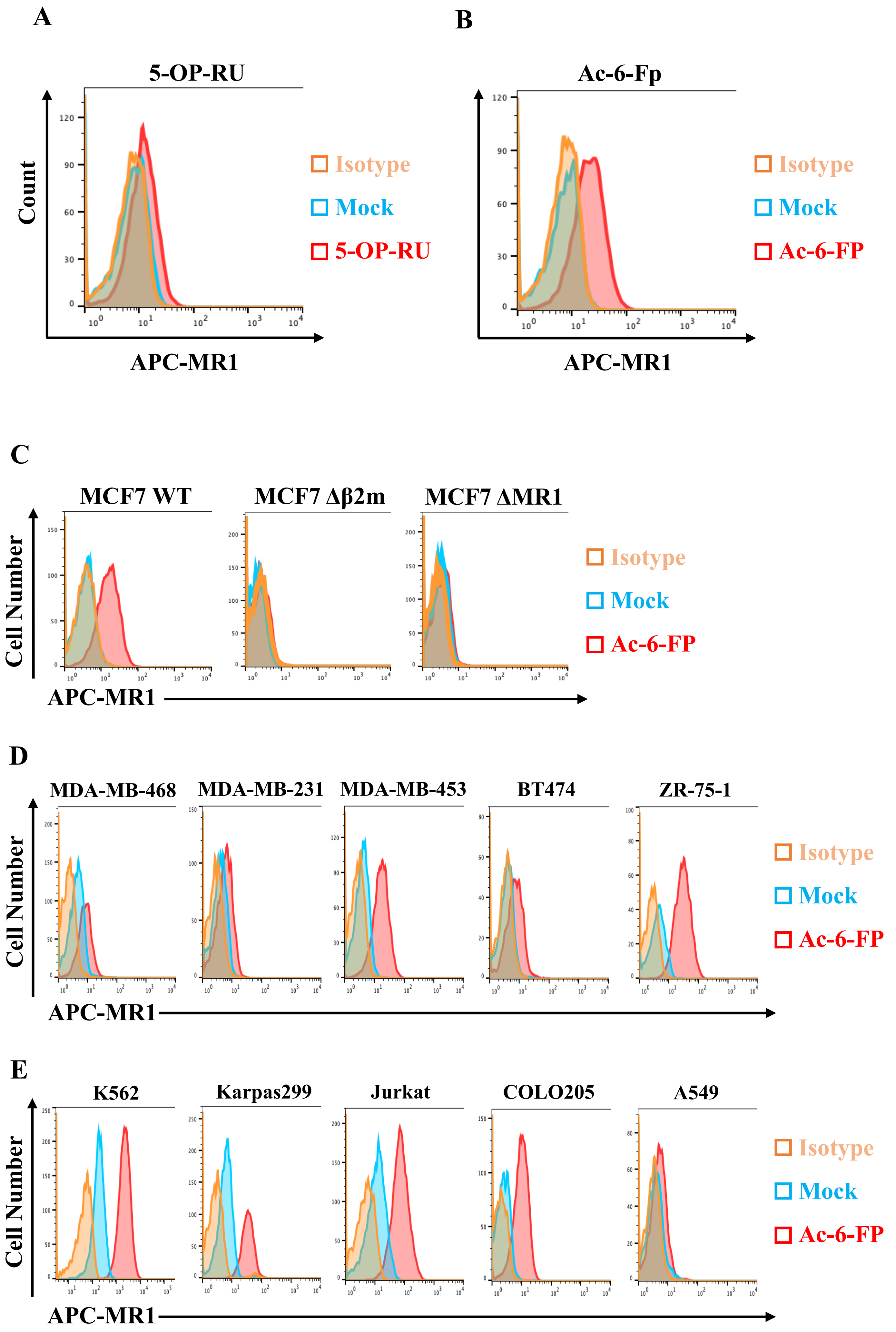

A
5-OP-RU
Count
APC-MR1
Isotype
Mock
5-OP-RU
B
Ac-6-Fp
APC-MR1
Isotype
Mock
Ac-6-FP
C
MCF7 WT
MCF7 Δβ2m
MCF7 ΔMR1
Cell Number
APC-MR1
Isotype
Mock
Ac-6-FP
D
MDA-MB-468
MDA-MB-231
MDA-MB-453
BT474
ZR-75-1
Cell Number
APC-MR1
Isotype
Mock
Ac-6-FP
E
K562
Karpas299
Jurkat
COLO205
A549
Cell Number
APC-MR1
Isotype
Mock
Ac-6-FP

Figure S4. Upregulation of MR1 surface expression on MCF7 cells in dose dependent manner. MCF7WT cells were treated with ***(A)*** 5-OP-RU (40µg ml^{-1}) and (*B*) Ac-6-FP (40µg ml^{-1}) overnight. The cells were stained with APC-conjugated anti-human/mouse/rat MR1 mAb and MR1 expression was analyzed by flowcytometry. Histograms showing the surface expression of MR1 on MCF7WT cells. (A single experiment is representative of three independent experiments). (*C*) Surface expression of MR1 on WT, β2m deleted and MR1 deleted MCF7 cells analyzed by flow cytometry after overnight stimulation with 4µg of Ac-6-FP. MR1 expression was analyzed by Flow cytometry. (A single experiment is representative of three independent experiments). (*D*) Human breast cancer cell lines were cultured overnight with or without Ac-6-FP. MR1-expression on these cells was analyzed by Flow cytometry. (*E*) Surface MR1 expression level on human tumor cell lines other than breast cancer cells.

Isotype: cells were stained with isotype control IgG, *Mock*: cells without incubating with Ac-6-FP/5-OP-RU, *Ac-6-FP*: cells treated with Ac-6-FP, *5-OP-RU*: cells treated with 5-OP-RU.

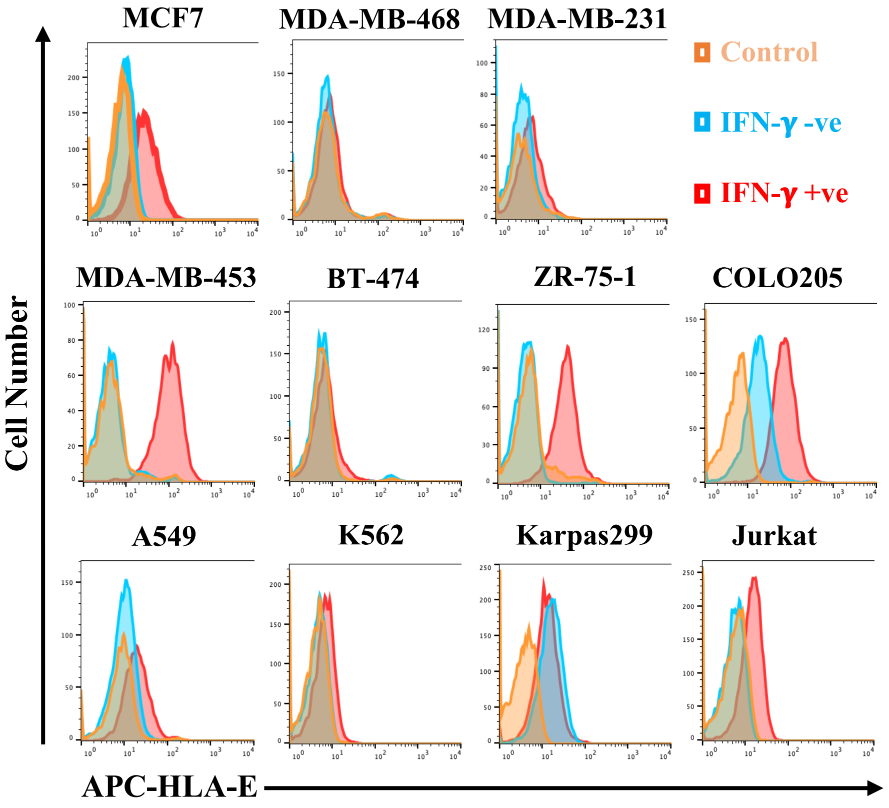

Figure S5. Detection of unconventional HLA-class I like molecule (HLA-E) on some human cancer cell lines. HLA-E expression was observed on human cancer cell lines from different origin. Cells were stimulated with hIFN-γ for 48 hours. Cells were stained with APC-conjugated anti-human HLA-E mAb and HLA-E expression on surface of cells was analyzed by flow cytometry. Histograms showing the surface expression of HLA-E on human cancer cell lines.

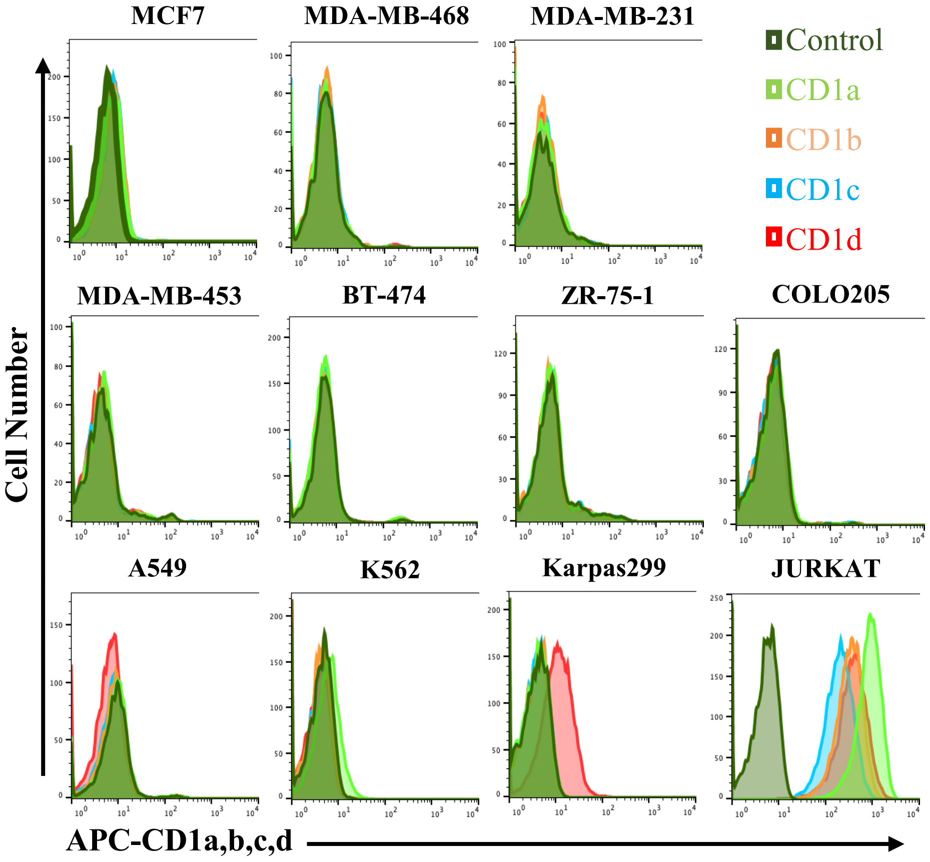

Figure S6. Surface Expression of CD1 a,b,c,d on some Human Cancer Cell Lines. Human cancer cell lines from different origin were stimulated with hIFN-γ for 48 hours and cells were stained with APC-conjugated anti-human CD1a, CD1b, CD1c and CD1d mAbs. Expression of CD1 a,b,c,d was analyzed by flowcytometry. Histograms showing the surface expression of CD1 a,b,c,d on some human tumor cell lines.

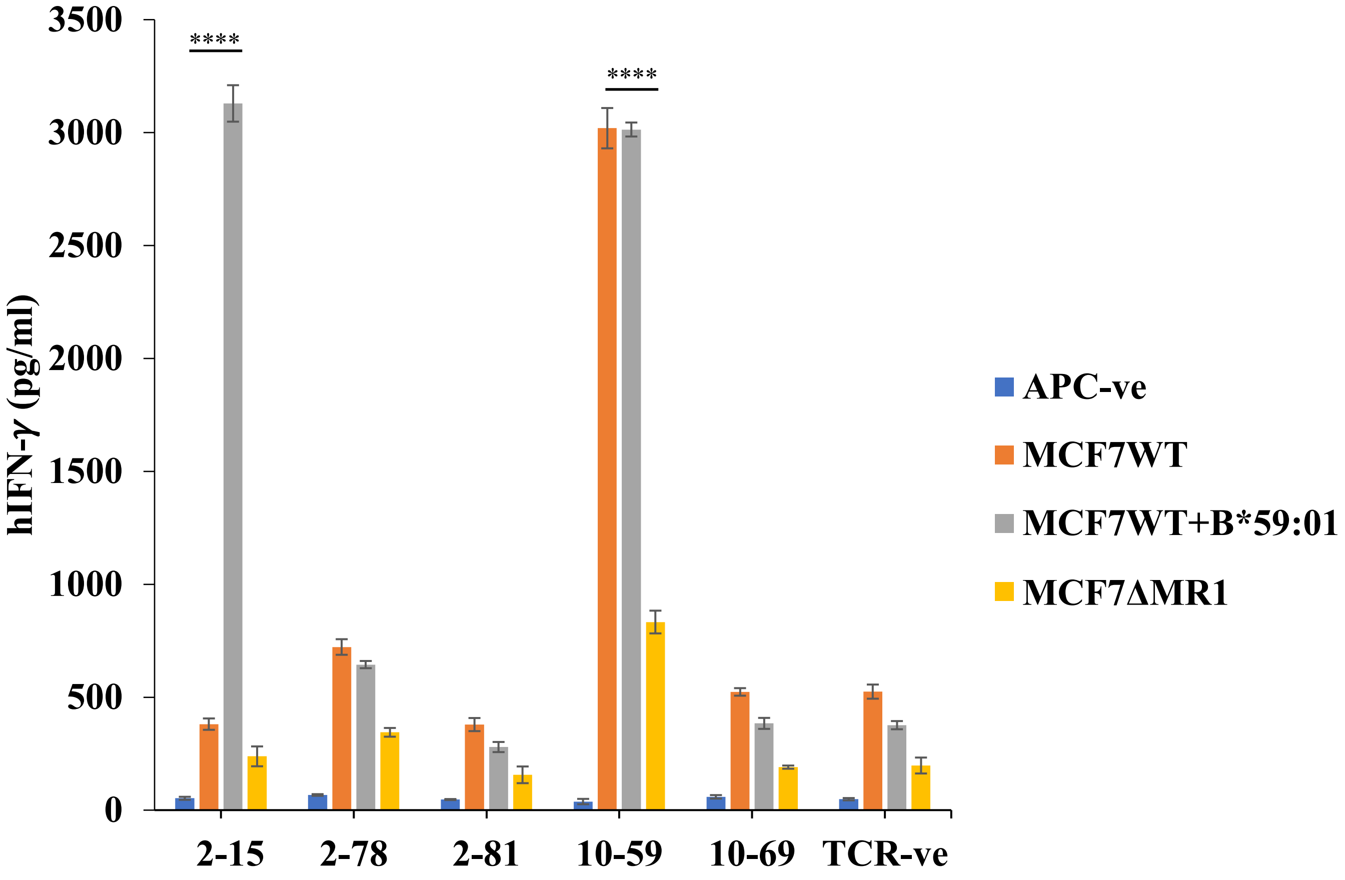

Figure S7. Reactivity of TCR-transduced human PBMCs derived from another donor. Human PBMCs were prepared from another donor, transduced with TCRs and cocultured with IFN-γ-stimulated MCF7 cells. IFN-γ-production in the supernatant was measured by ELISA in triplicate. TCR negative (TCR-ve) T cells were used as a negative control. A single experiment is representative of three independent experiments. Mean and SD values from technical triplicate cultures are indicated. Unpaired, two-tailed t tests were performed (*P<0.05, **P<0.01, ***P<0.001, ****P<0.0001 and ns, not significant).

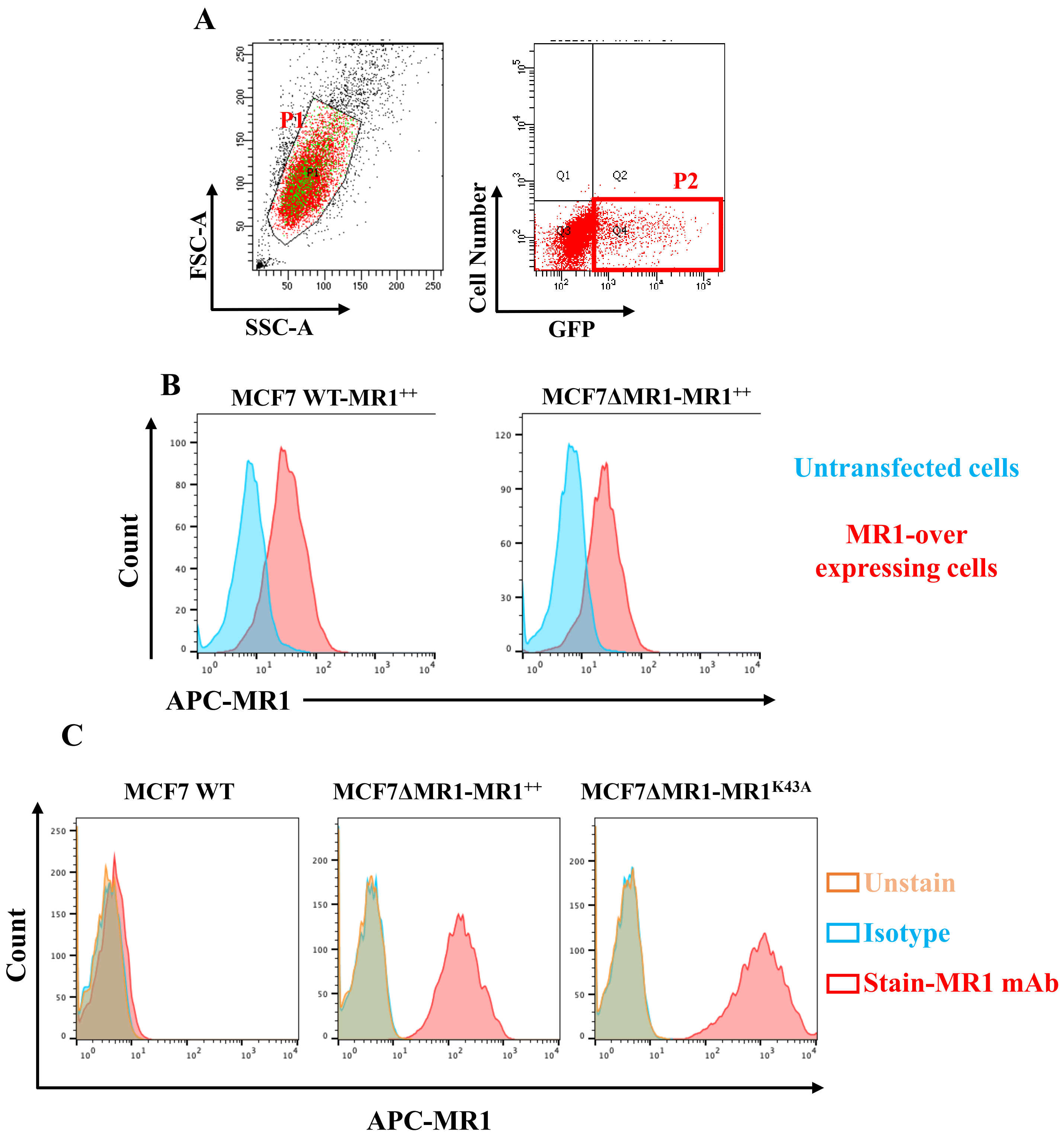

Figure S8. Overexpression of MR1 on MCF7 cells. MR1-expression vector containing IRES-GFP was transduced into MCF7 cells by using Piggybac system. After 48 hours, cells were sorted as GFP+ve cells. (*A*) shows gating strategy for sorting MR1expressed MCF7 cells. The gate of live MCF7 cells was set using forward scatter and side scatter (P1) and the percentages of GFP+ cells were indicated in P2. (*B*) MR1 expression was analyzed on MR1-over expressing MCF7 cells by staining cells with APC-conjugated anti-human/mouse/rat MR1 mAb. Un-transfected cells were stained with APC-MR1 mAb as negative controls. Histograms showing MR1 expression on MCF7 cells after sorting. (*C*) MR1 expression was observed on MCF7 cells after K43A alteration. Cells were stained with APC-conjugated anti-human/mouse/rat MR1 mAb. Histograms are showing MR1 expression on MCF7 cells after re-expression of MR1 gene and K43A alteration.

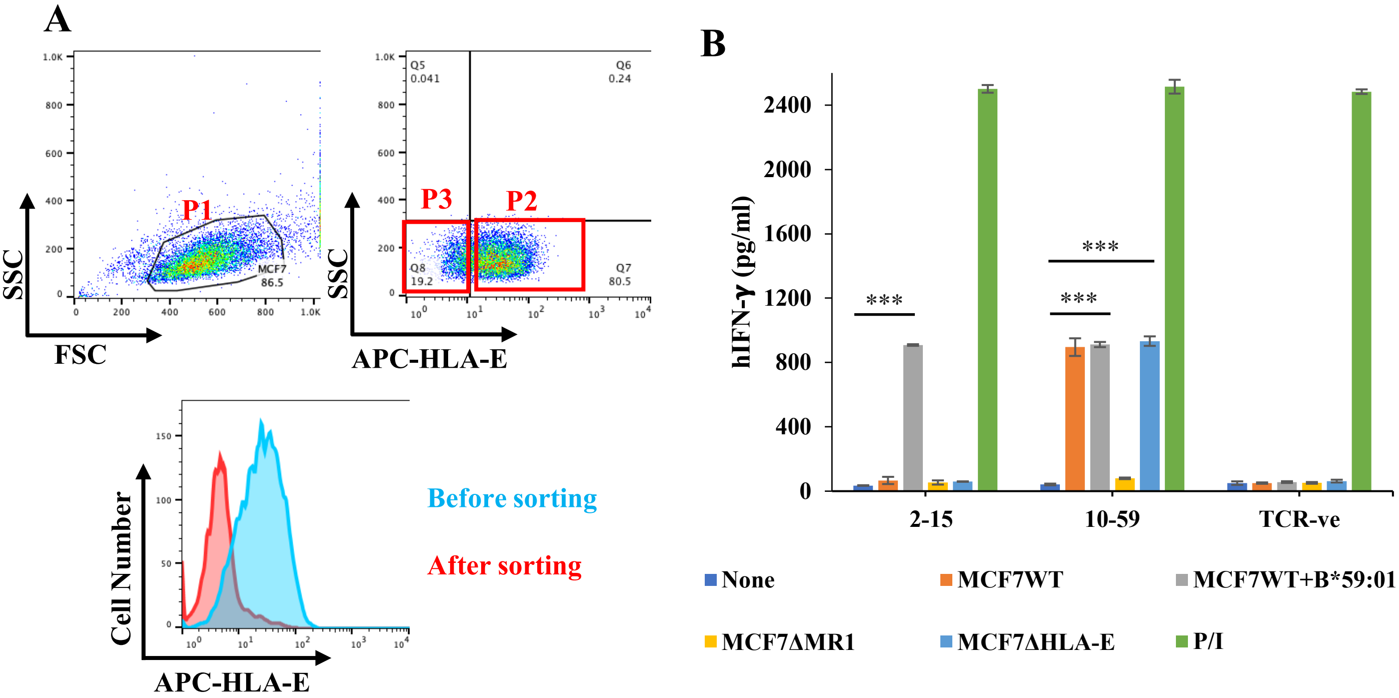

Figure S9. Reactivity of TCR10-59 on HLA-E knocked out MCF7 cells. (*A*) After knocking down HLA-E gene, hIFN-γ treated MCF7 cells were stained with APC- conjugated mAbs to analyze cell surface expression of HLA-E. The gate of live MCF7 cells was set using forward scatter and side scatter (P1) and the percentages of APC+ cells were indicated in P2. We sorted APC-ve cells (P3) for generation of MCF7ΔHLA-E cells. Histograms showing HLA-E expression on MCF7 cells before and after sorting stained with APC-conjugated mAb. (*B*) TCR-expressing T cells were co-cultured with IFN-γ-stimulated MCF7 cells. IFN-γ production was measured by ELISA. For negative control, TCR-ve T cells were used. T cells were stimulated with PMA + Ionomycin and used as positive control. (A single experiment is representative of 3 independent experiments). Mean and SD values from technical triplicates cultures are indicated. Unpaired, two-tailed t-tests were performed (*P<0.05,**P<0.01, ***P<0.001 and ns, not significant).

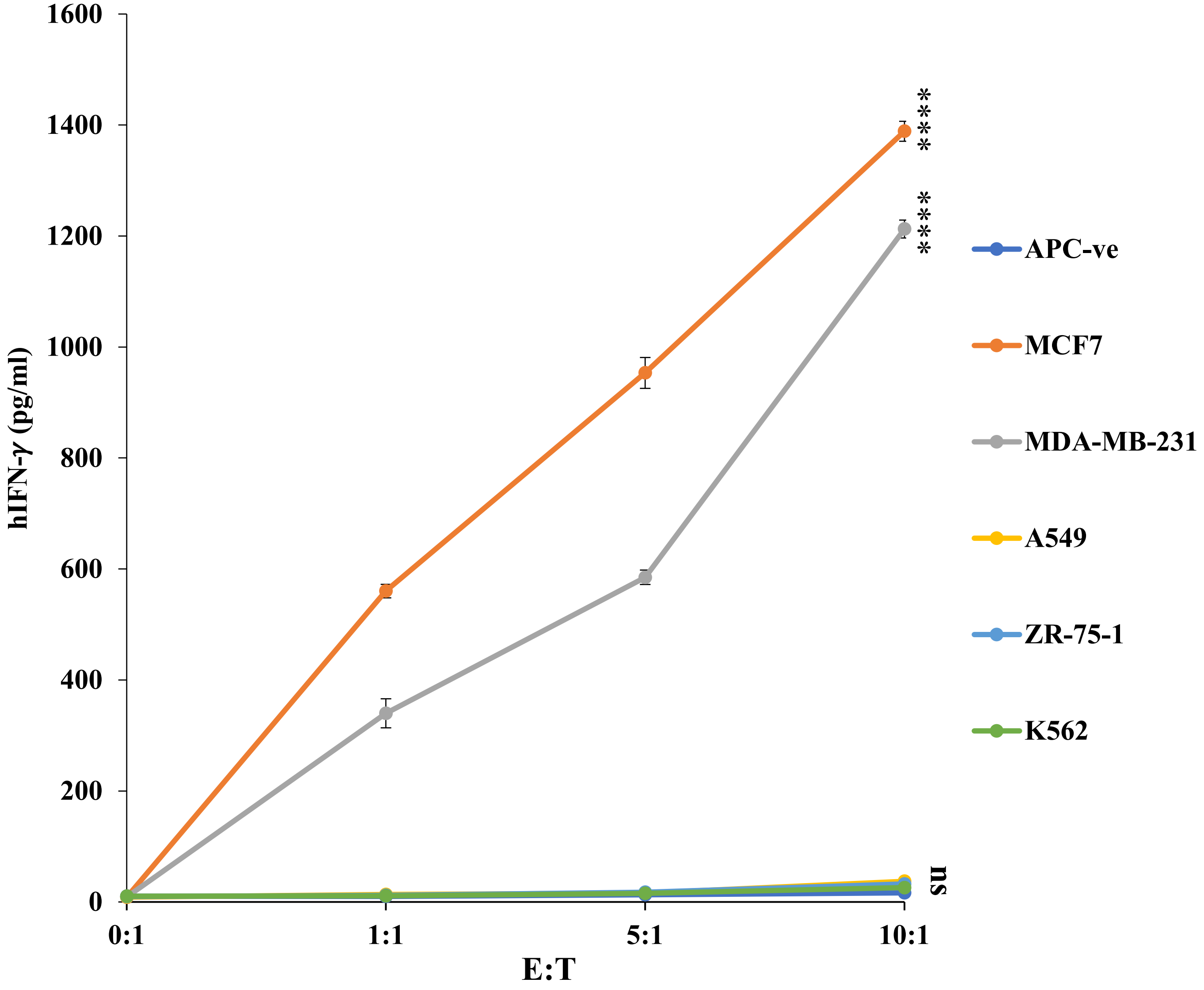

Figure S10. Dose response of TCR10-59-expressing PBMCs against target cells. TCR10-59 was retrovirally transduced into PBMCs and cocultured with IFN-γ-stimulated MCF7, MDA-MB-231, A549, ZR-75-1 and K562 cell at different effector-to-target (E:T) ratios. APC-ve cultures were used as a negative control. ELISA was performed to analyze E:T responses in the supernatant by measuring IFN-γ secretion. A single experiment is representative of two independent experiments with similar results. Mean and SD values from technical triplicate cultures are indicated. Unpaired, two-tailed t tests were performed (*P<0.05, **P<0.01, ***P<0.001, ****P<0.0001 and ns, not significant).

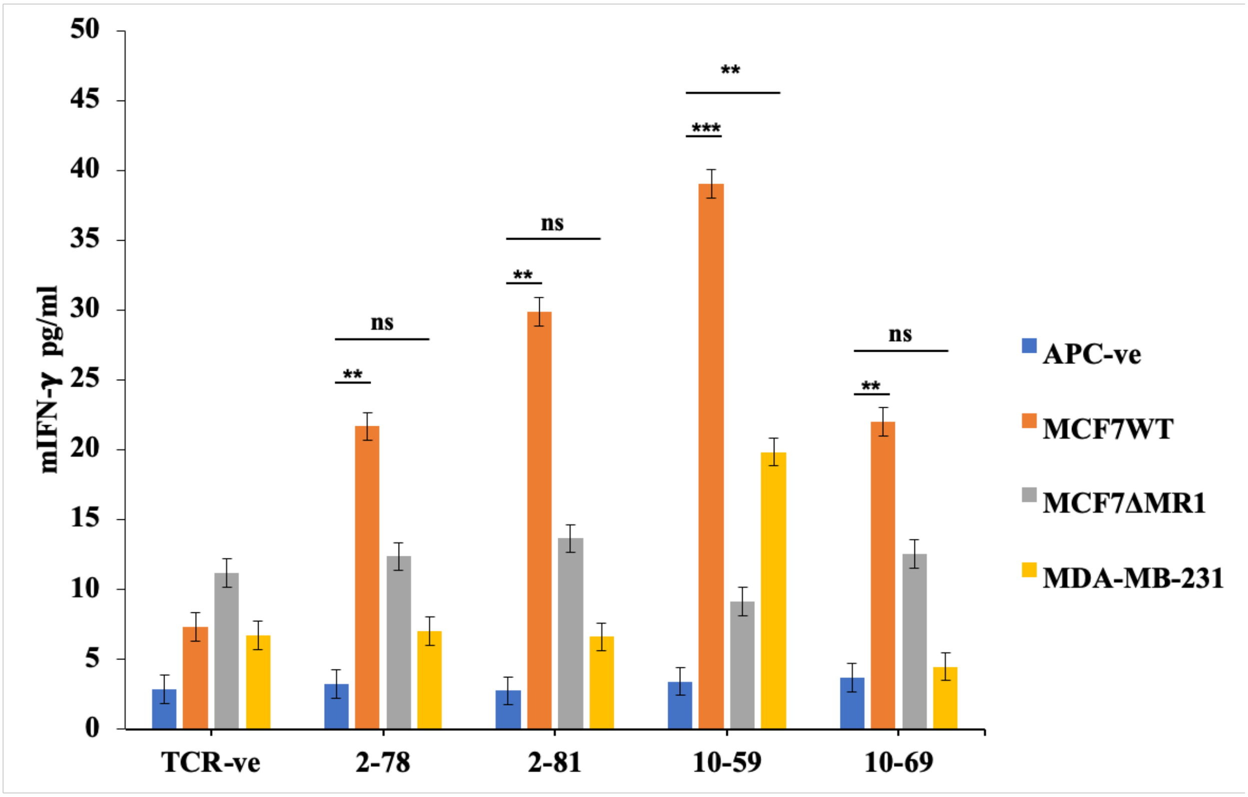

Figure S11. Breast cancer cell-specific reactivity of MR1-restricted TCRs. TCRs expressing-Mouse splenic T cells were co-cultured with IFN-γ-stimulated MCF7WT, MCF7ΔMR1 and MDA-MB-231 cells. The reactivity against MCF7 cells was measured by mouse IFN-γ ELISA in triplicates. TCR negative (TCR-ve) T cells were used as negative control. (A single experiment is representative of three independent experiments). Mean and SD values from technical triplicate cultures are indicated. Unpaired, two-tailed t tests were performed (*P<0.05, **P<0.01, ***P<0.001 and ns, not significant).

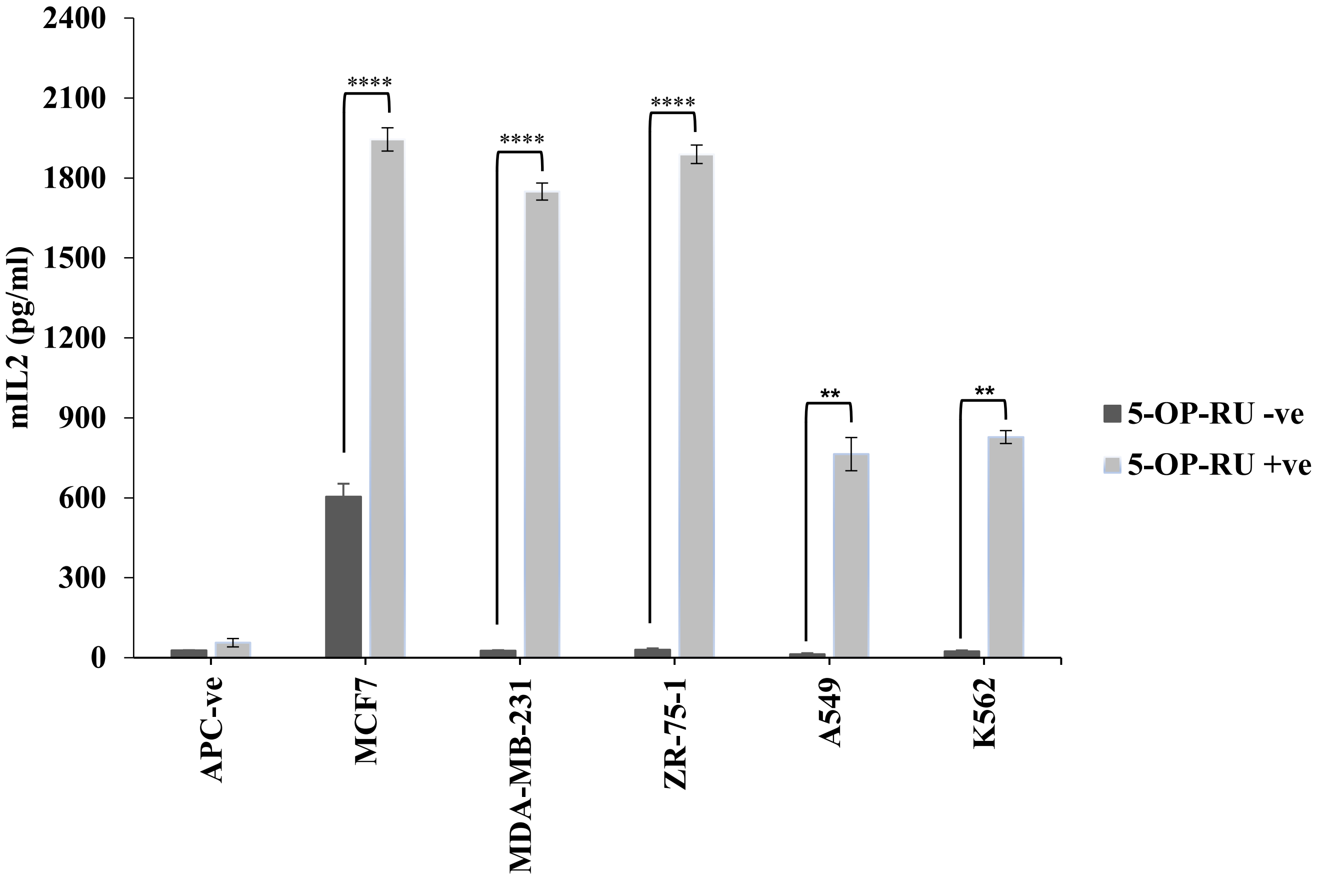

Figure S12. Effect of 5-OP-RU on MAIT cell stimulation against human cancer cell lines. TCR 2-78 -expressing BW cells were cocultured with various IFN-γ-stimulated human cancer cell lines for 24 hours with/without 5-OP-RU (125nM). MCF7 cells were used as positive control. ELISA was performed to measure mouse IL-2 secretion in the supernatants. The experiment was performed two times with similar results. Mean and SD values from technical triplicate cultures are indicated. Unpaired, two-tailed t tests were performed (*P<0.05, **P<0.01, ***P<0.001, ****P<0.0001 and ns, not significant).

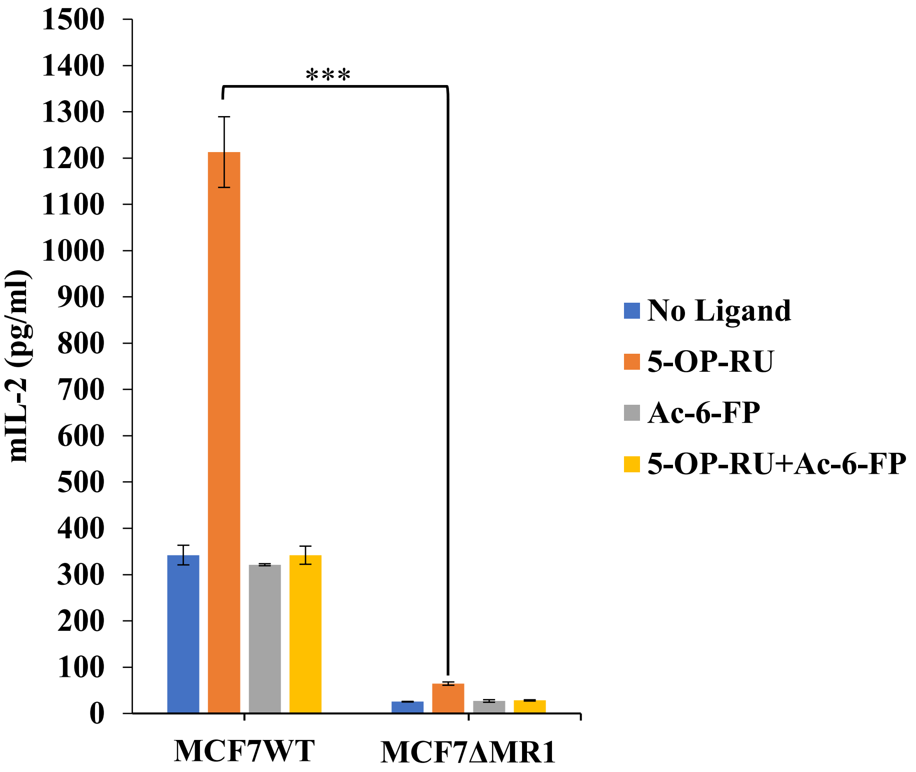

Figure S13. Effect of 5-OP-RU on MR1-mutant MCF7 cells. TCR 2-78 -expressing BW cells were cocultured with IFN-γ-stimulated MCF7 cells for 24 hours with/without 5-OP-RU (125nM) and Ac-6-FP (20 µg $ml^{-1)}$.ELISA was performed to measure mouse IL-2 secretion in the supernatants. The experiment was performed two times with similar results. Mean and SD values from technical triplicate cultures are indicated. Unpaired, two-tailed t tests were performed (*P<0.05, **P<0.01, ***P<0.001 and ns, not significant).

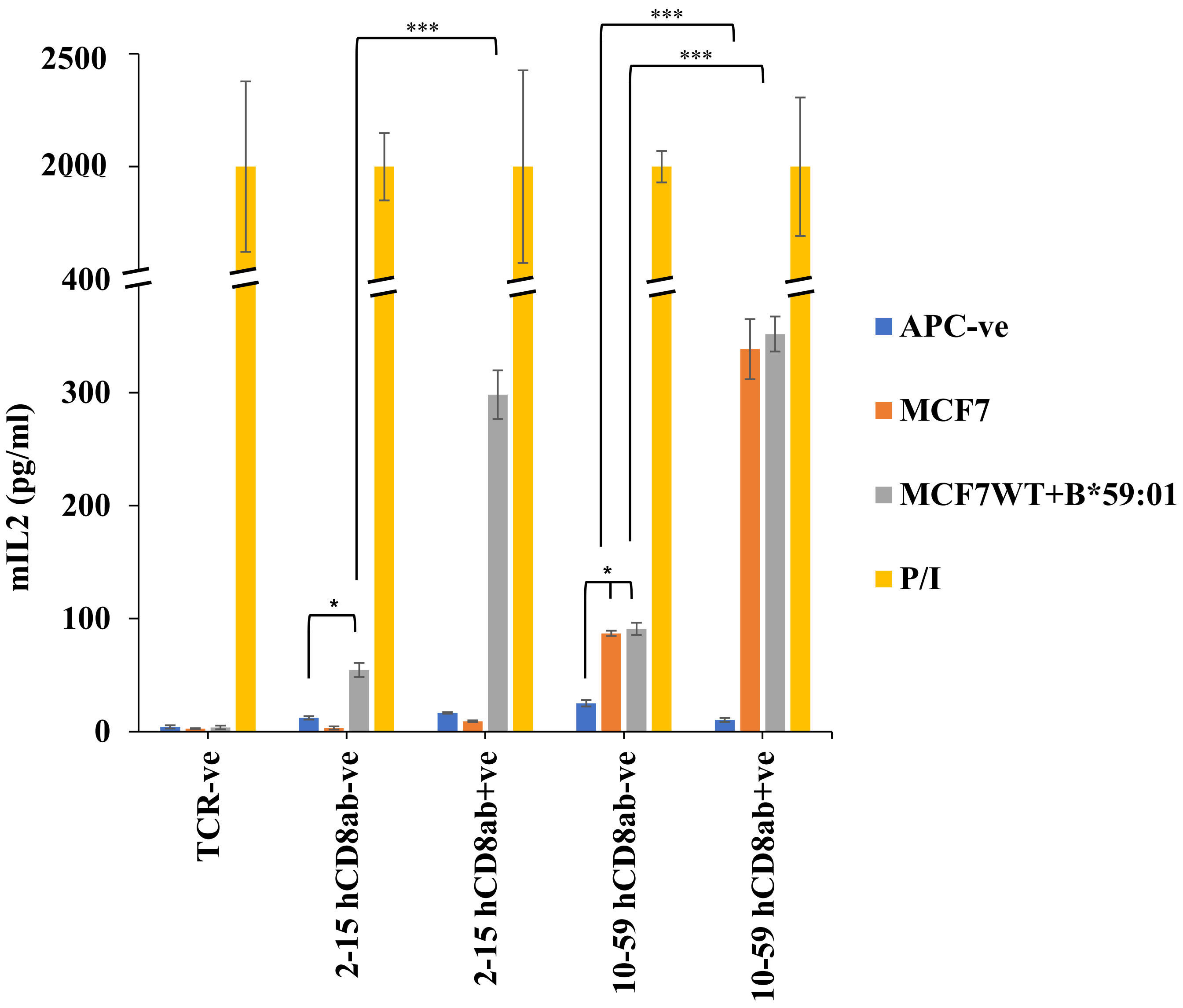

Figure S14. hCD8ab enhances reactivity of T cells. TCR2-15 (Conventional TCR) and TCR10-59 (MR1-restricted TCR)-expressing BW with/without hCD8ab cells were cocultured with IFN-γ-stimulated MCF7WT and MCF7WT+B*59:01 cells. Responses were assessed by analyzing IL-2-production in the supernatant. T cells were stimulated with PMA + Ionomycin and used as positive control. The experiment was performed two times with similar results. Mean and SD values from technical triplicate cultures are indicated. Unpaired, two-tailed t tests were performed (*P<0.05, **P<0.01, ***P<0.001 and ns, not significant).

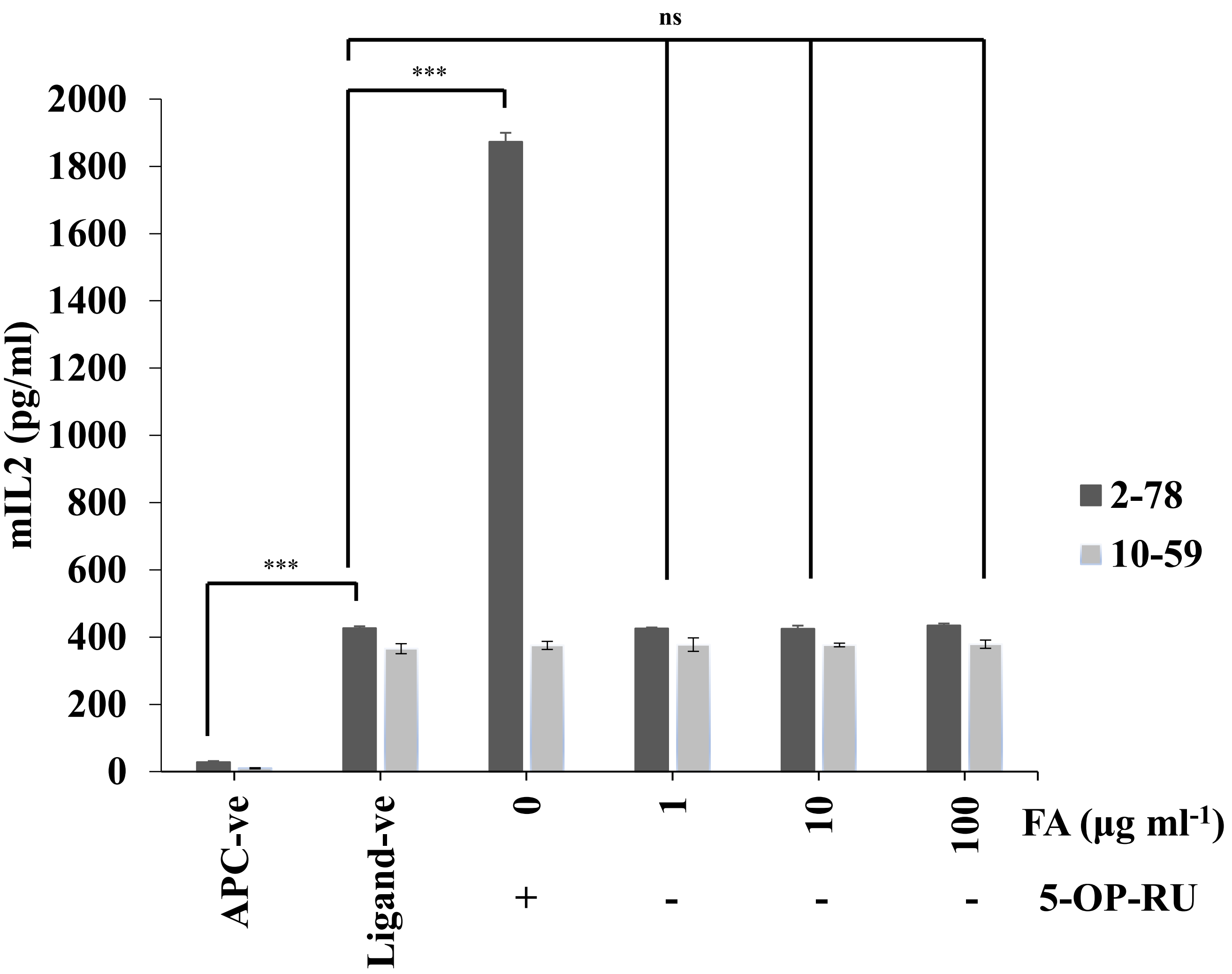

Figure S15. Effect of Folic Acid on stimulation of MR1T cells. TCR2-78 and TCR10-59-expressing BW cells were cocultured with IFN-γ-stimulated MCF7WT cells for 24 hours. APC-ve denotes cultures without MCF7 cells. Folic acid (FA) was also added to the culture at different doses. 5-OP-RU (125nM) was also added to the culture where indicated. Responses were assessed by analyzing IL-2-production in the supernatant. The experiment was performed two times with similar results. Mean and SD values from technical triplicate cultures are indicated. Unpaired, two-tailed t tests were performed (*P<0.05, **P<0.01, ***P<0.001 and ns, not significant).

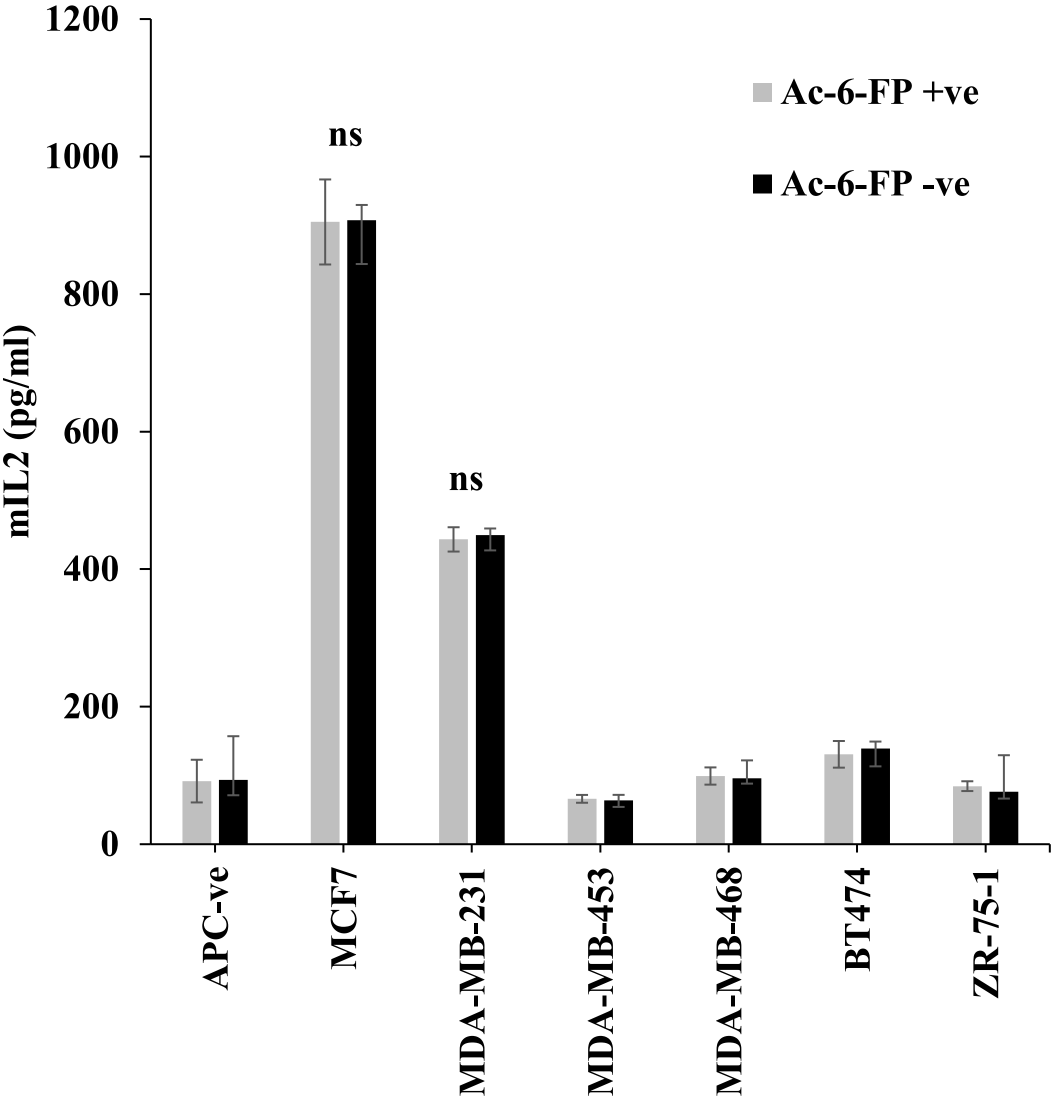

Figure S16. Effect of Ac-6-FP on reactivity of TCR10-59. TCR10-59 (MR1-restricted TCR)-expressing BWhCD8ab cells were cocultured with IFN-γ-stimulated breast cancer cell lines (MCF7WT, MDA-MB-231, MDA-MB-453, MDA-MB-468, BT474, ZR-75-1) in the presence or absence of Ac-6-FP. Responses were assessed by analyzing IL-2-production in the supernatant. APC-ve cultures were used as negative control. The experiment was performed two times with similar results. Mean and SD values from technical triplicate cultures are indicated. Unpaired, two-tailed t tests were performed (*P<0.05, **P<0.01, ***P<0.001 and ns, not significant).

Table S1. Summary of MAIT cells and MR1 T cells reactivity.

TCR	Bacterial Ag or 5-OP-RU dependency	Inhibition by 6-FP or Ac-6-FP	Inhibition by MR1 Ab	Dependency on K43 residue of MR1
MAIT cells	+	+	+	+
Crowther et al. [1]	−	+	+	+
Lepore et al. [2]	−	+	+	−
Our TCRs	−	−	−	+

References

1. Crowther, M.D.; Dolton, G.; Legut, M.; Caillaud, M.E.; Lloyd, A.; Attaf, M.; Galloway, S.A.E.; Rius, C.; Farrell, C.P.; Szomolay, B.; et al. Genome-wide CRISPR–Cas9 screening reveals ubiquitous T cell cancer targeting via the monomorphic MHC class I-related protein MR1. *Nat. Immunol.* **2020**, *21*, 178–185.

2. Lepore, M.; Kalinichenko, A.; Calogero, S.; Kumar, P.; Paleja, B.; Schmaler, M.; Narang, V.; Zolezzi, F.; Poidinger, M.; Mori, L.; et al. Functionally diverse human T cells recognize non-microbial antigens presented by MR1. *Elife* **2017**, *6*, e24476.
